# Mobile Phone–Supported Physiotherapy for Frozen Shoulder: Feasibility Assessment Based on a Usability Study

**DOI:** 10.2196/rehab.7085

**Published:** 2017-07-20

**Authors:** Thomas Stütz, Gerlinde Emsenhuber, Daniela Huber, Michael Domhardt, Martin Tiefengrabner, Gertie Janneke Oostingh, Ulrike Fötschl, Nicholas Matis, Simon Ginzinger

**Affiliations:** ^1^ SmartHealthCheck Project Department of Multimedia Technology University of Applied Sciences Salzburg Puch / Salzburg Austria; ^2^ SmartHealthCheck Project Department of Physiotherapy University of Applied Sciences Salzburg Puch / Salzburg Austria; ^3^ SmartHealthCheck Project Department of Biomedical Sciences University of Applied Sciences Salzburg Puch / Salzburg Austria; ^4^ Ambulatory Shoulder Care Department of Orthopedics and Traumatology University Hospital Salzburg (SALK) Salzburg Austria

**Keywords:** telemedicine, mobile health, mHealth, frozen shoulder, adhesive capsulitis, physiotherapy (techniques), home health aides, mobile phone

## Abstract

**Background:**

Patients with frozen shoulder show limited shoulder mobility often accompanied by pain. Common treatment methods include physiotherapy, pain medication, administration of corticosteroids, and surgical capsulotomy. Frozen shoulder often lasts from months to years and mostly affects persons in the age group of 40 to 70 years. It severely reduces the quality of life and the ability to work.

**Objective:**

The objective of this study was to evaluate the feasibility of a mobile health (mHealth) intervention that supports patients affected by “stage two” frozen shoulder. Patients were supported with app-based exercise instructions and tools to monitor their training compliance and progress. These training compliance and progress data supplement the patients’ oral reports to the physiotherapists and physicians and can assist them in therapy adjustment.

**Methods:**

In order to assess the feasibility of the mHealth intervention, a pilot study of a newly developed app for frozen shoulder patients was conducted with 5 patients for 3 weeks. The main function of the app was the instruction for exercising at home. Standardized questionnaires on usability such as System Usability Scale (SUS) and USE (Usefulness, Satisfaction, and Ease of use), and Technology Acceptance Model-2 (TAM-2) were completed by the study participants at the end of the study. Additionally, a nonstandardized questionnaire was completed by all patients. The correctness of the exercises as conducted by the patients was assessed by a physiotherapist at the end of the study. The mobility of the shoulder and pain in shoulder movement was assessed by a physiotherapist at the start and the end of the study.

**Results:**

The pilot study was successfully conducted, and the app was evaluated by the patients after 3 weeks. The results of the standardized questionnaires showed high acceptance (TAM-2) and high usability (SUS) of the developed app. The overall usability of the system as assessed by the SUS questionnaire was very good (an average score of 88 out of 100). The average score of the TAM-2 questionnaire on the intention to further use the app was 4.2 out of 5, which indicated that most patients would use the app if further available. The results of the USE questionnaires highlighted that the patients learned how to use the app easily (an average score of 4.2 out of 5) and were satisfied with the app (an average score of 4.7 out of 5). The frequency of app usage and training was very high based on patient reports and verified by analysis of the usage data. The patients conducted the exercises almost flawlessly.

**Conclusions:**

Our results indicate the feasibility of the mHealth intervention, as the app was easy to use and frequently used by the patients. The app supported the patients’ physiotherapy by providing clear exercising instructions.

## Introduction

Shoulder stiffness is a condition associated with the restriction of active and passive range of motion. A variety of conditions are classified according to underlying pathologies, which could be intrinsic (pathology inside the joint), extrinsic (pathology outside the joint), and systemic (related to systemic diseases) in nature. All these conditions are summarized under “secondary shoulder stiffness.” In contrast to these, the onset of primary idiopathic shoulder stiffness, also known as frozen shoulder, occurs without any apparent reason. The incidence for a frozen shoulder is reported to be 2% to 3.5% in the general population [1,2]; people in the age group of 40 to 70 years are affected more frequently [1,2]. Additionally, 10% to 36% of diabetics are affected by frozen shoulder at least once in their lifetime [3,4]. The occurrence of thyroid diseases is also linked with a fourfold increase in the risk of developing frozen shoulder [3]. In the diagnostic classification systems, International Classification of Diseases, Tenth Revision (ICD-10) and ICD-9-CM (Ninth Revision, Clinical Modification), frozen shoulder is included in the class “adhesive capsulitis” and no distinction is made between primary, idiopathic, and secondary causes. However, the term “adhesive capsulitis” does not describe the pathological process accurately [5] and thus, the term “frozen shoulder” is used consistently in our work to refer to primary idiopathic adhesive capsulitis. Frozen shoulder commonly lasts 2 to 3 years, yet the course of a frozen shoulder can vary greatly and symptoms may persist [6]. The process of a frozen shoulder is divided into three stages. It starts with a painful freezing stage characterized by an inflammatory process in the synovia and the capsule of the shoulder joint. The freezing stage is followed by a frozen stage, in which pain slowly subsides, but restriction in active and passive mobility develops. Abduction and external rotation are the most affected directions of movement, followed by internal rotation and flexion. This condition can last for several months up to several years. In the final stage, the thawing stage, mobility improves, yet for up to half of the patients limitations in mobility remain to some degree [7].

The annual treatment cost for a patient affected by frozen shoulder is estimated between $7000 and $8000 [3]. These treatment costs do not include the costs associated with the loss of productivity due to work disability and sick leaves. The negative effect of the patients’ reduction in quality of life is not considered by costs at all. While frozen shoulder is a common disease with a large morbidity, high quality evidence for successful treatment methods is still missing [1,8-15]. Most common treatments are pain medication, physiotherapy, and surgery [16].

Physiotherapy, including mobilization and strength exercises, is a common treatment in the early painful phase as well as the resolution phase [16]. In most cases, these exercises are performed at home and not under the constant supervision of a physiotherapist, due to financial and time constraints. Exercising at home presents two difficulties for patients: training compliance and exercise correctness. Training frequency and duration at home is not maintained as intended. Noncompliance rates as high as 70% have been reported [17]. In a previous study, only 8 of 20 patients were reported to be fully compliant to physiotherapy during therapy sessions, and only 7 of 20 were reported fully compliant after the therapy ended [18]. A main factor for compliance is the successful inclusion of exercising into daily life [18]. The other main issue of home-exercise–based physiotherapy is that the majority of patients were not performing exercises at home correctly after 2 weeks of receiving their initial instructions [19]. Compliance and exercise correctness can be tackled by motivational tools and better instructions that are accessible at home. Mobile phones have become common and, therefore, a mobile phone app aiming to support patients with frozen shoulder through motivational tools and improved home instructions can be a viable contribution in the treatment of this disease.

The aim of this study was to conduct a pilot study to evaluate the feasibility of a mobile phone–based mobile health (mHealth) intervention for frozen shoulder.

The main research question was whether the mHealth intervention was feasible, that is, whether the app could be successfully employed in a field study. Evaluated measures for success were app usability, training compliance, and exercise correctness.

The organization of the study follows the guidelines for evaluation studies in health informatics [20].

## Methods

### Study Context

#### Organizational Setting

The initiative to develop an app was taken by the head of shoulder surgery at the University Hospital Salzburg (Salzburger Landeskliniken, Universitätsklinikum Salzburg, SALK), Department of Orthopedics and Trauma Surgery of the Paracelsus Medical University, Salzburg, which is a level one trauma center. The app was developed and tested at the Department of Multimedia Technology at the Salzburg University of Applied Sciences (SUAS). The study was conducted at the educational facility of the Department of Physiotherapy of the SUAS, which is located at the main facility of the SALK.

#### A Mobile Phone App to Support Patients With Frozen Shoulder

The app for frozen shoulder patients was developed in a co-creation process, which included a training mode with detailed instructions on exercise conduct, an exercise calendar, and a mobile phone sensor–based mobility measurement (see Multimedia Appendix 1). The exercises were demonstrated by means of a three-dimensional (3D) avatar, which performed the exercises as intended.

The Unity3D game engine was used to implement the app. The exercises were first recorded with a 3D capturing system (OptiTrack) and on video. The OptiTrack recordings and videos were used by a 3D modeler to create accurate animations of the exercises. Several interface concepts were tested and evaluated by the authors and their colleagues (see Acknowledgments). App development was an iterative process of analysis, conceptualizing, and prototyping in a focus group. This prototype was evaluated in a focus group consisting of 8 potential patients typical for the target group and 5 physiotherapists. The final prototype for the pilot study is explained in detail in the following sections.

**Figure 1:**
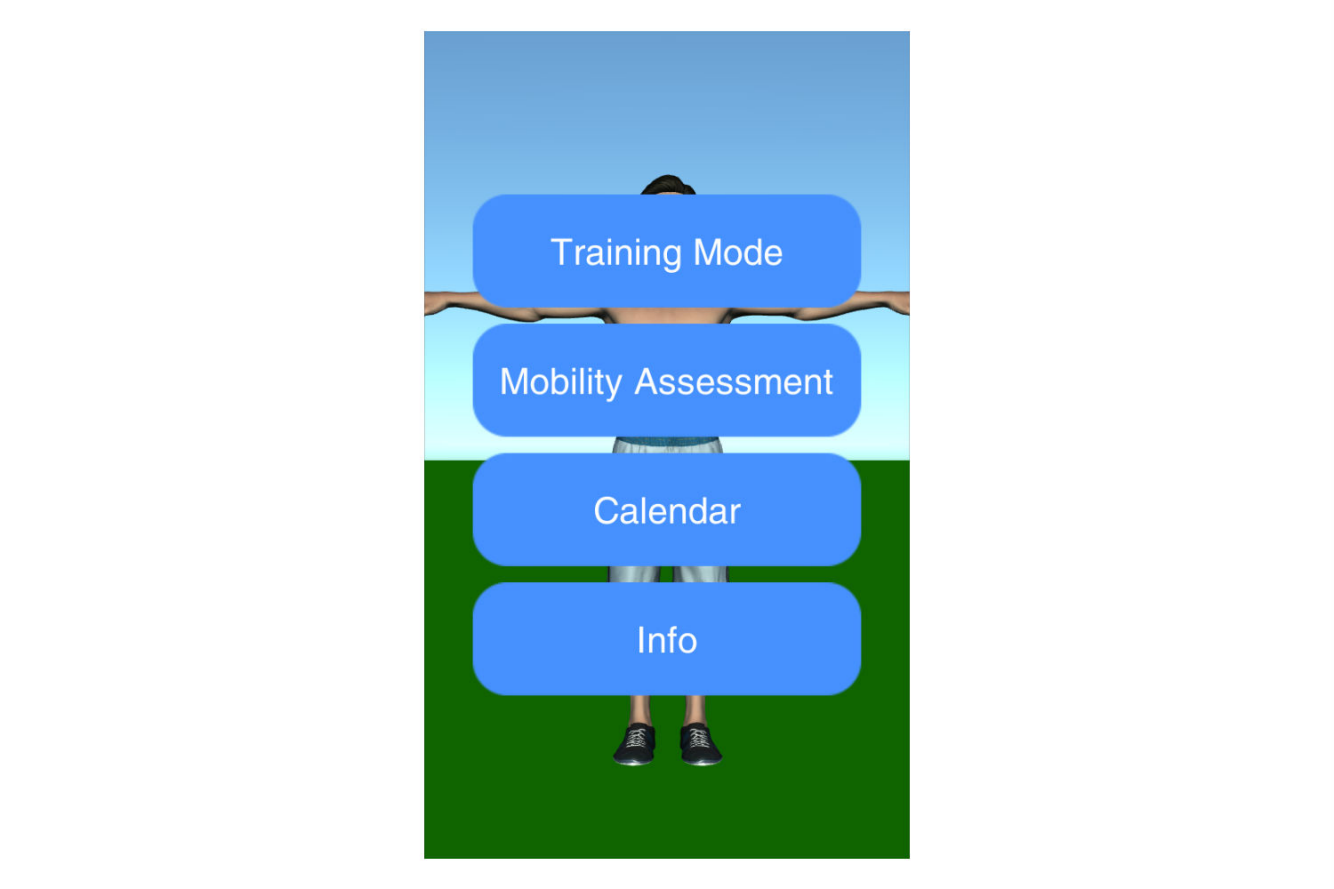
Start menu view.

The main screen of the frozen shoulder app for the patients in the pilot study had 4 buttons to access four functions (see Figure 1):

1. Training Mode
2. Mobility Assessment
3. Calendar
4. Info

**Figure 2:**
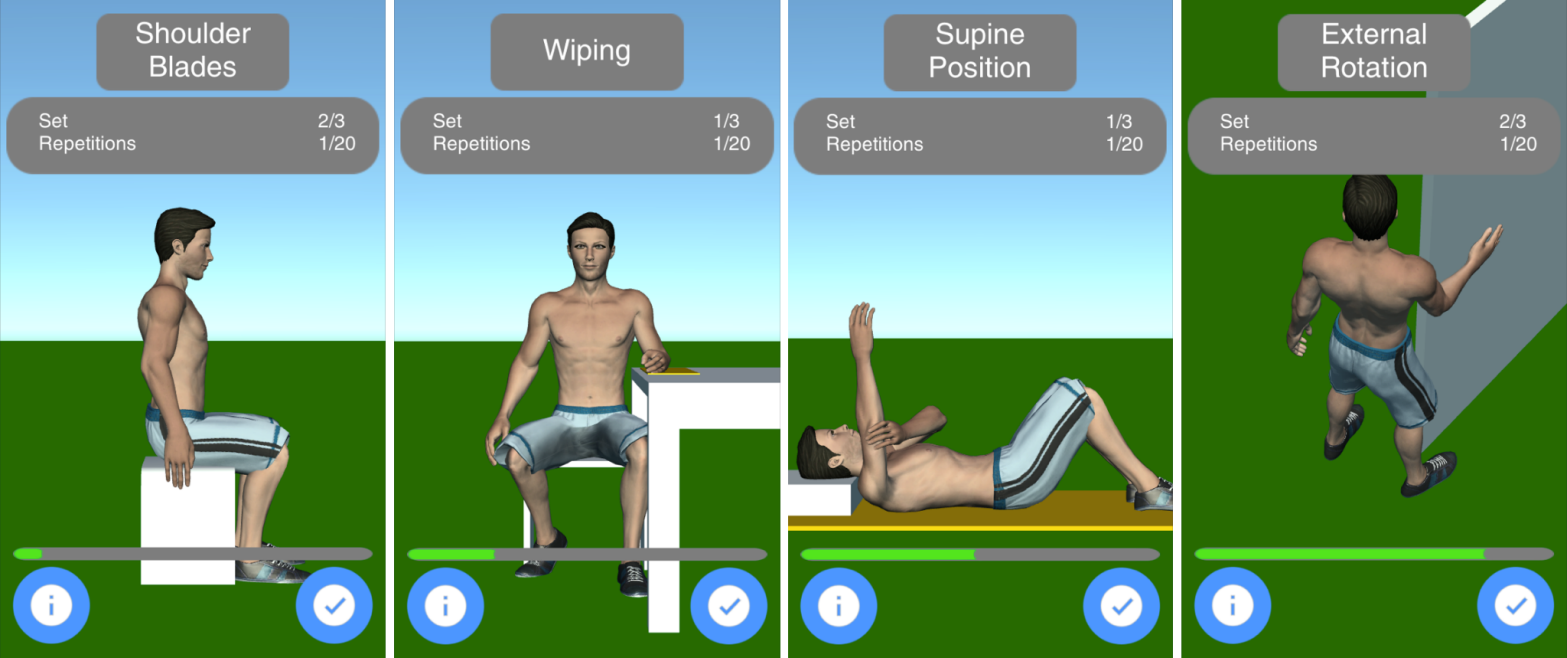
Training mode.

The training mode included instructions for four exercises (selected by a team of physiotherapists and the physician), which are shown in Figure 2. In the first exercise, the shoulders are moved up and down (see first screen of Figure 2). In the second exercise, the affected arm is mobilized on a table (see second screen of Figure 2). For the third exercise, the patient is lying down and laterally moving the affected arm, while the other arm is used for support (see third screen of Figure 2). The fourth exercise involves the use of doorframe for external rotational stretching (see fourth screen of Figure 2). The app recommends three sets with 20 repetitions for each exercise.

**Figure 3:**
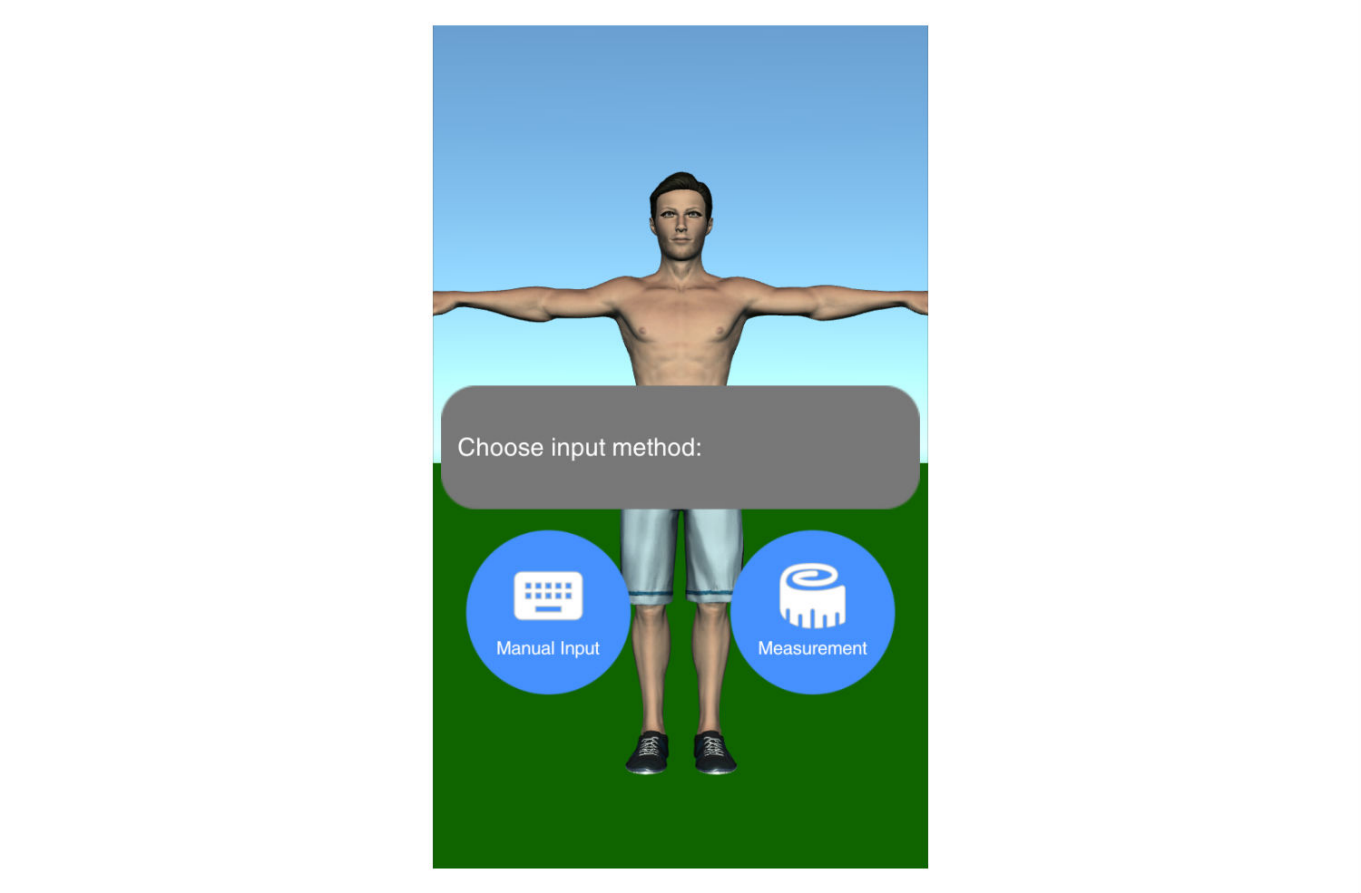
Start screen of mobility assessment.

**Figure 4:**
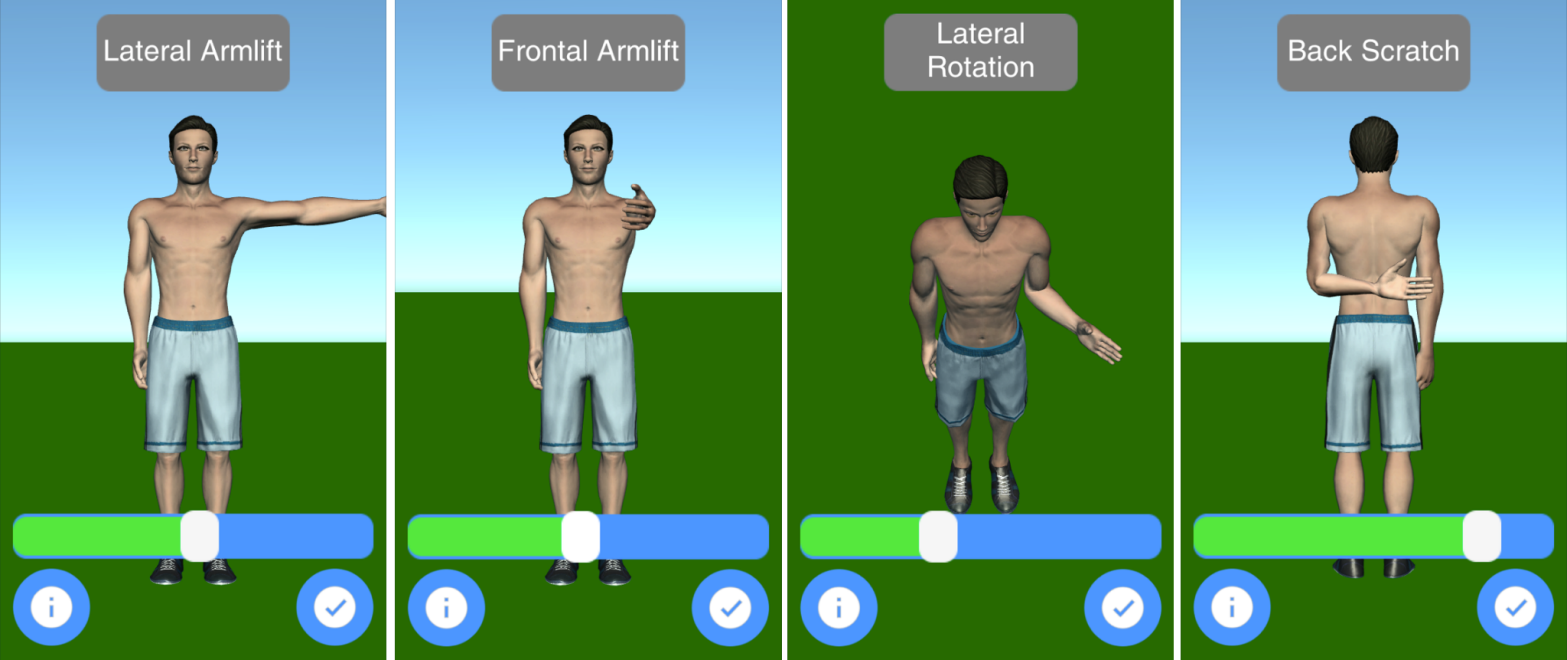
Mobility assessment.

**Figure 5:**
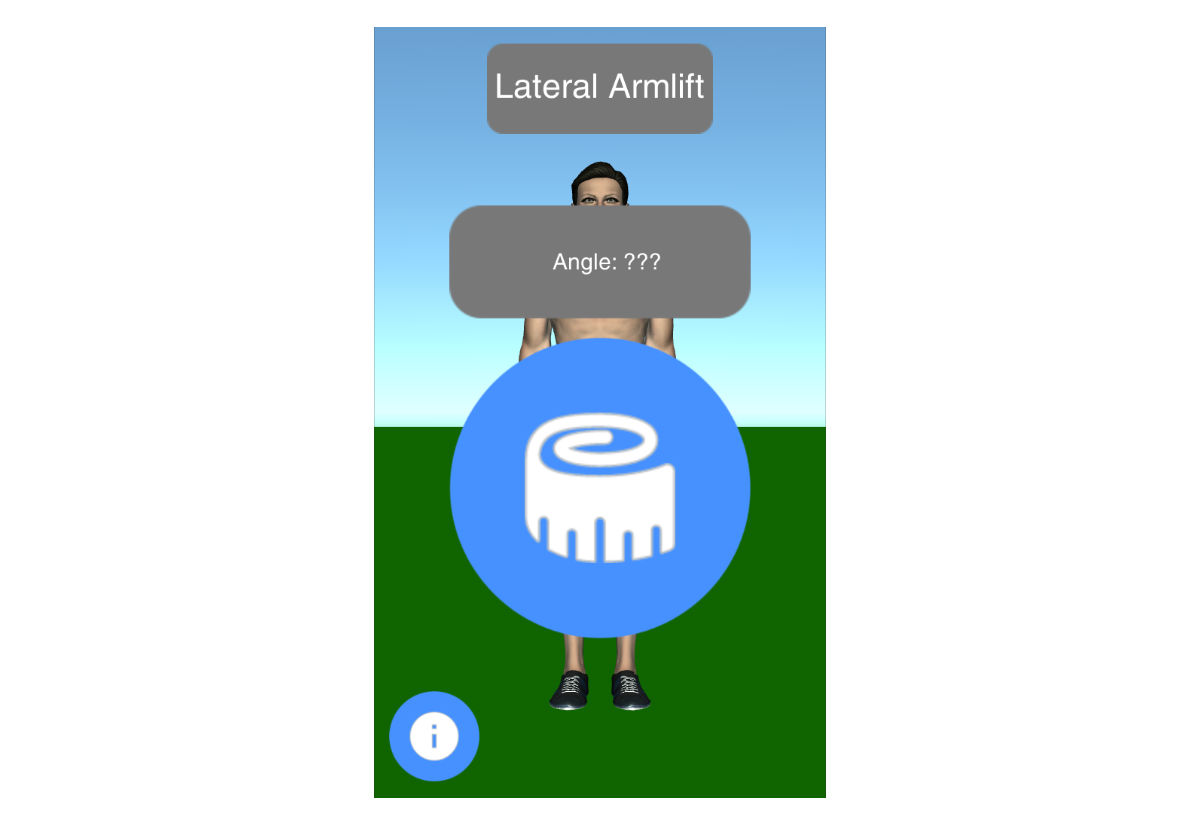
Start screen sensor-based mobility assessment.

**Figure 6:**
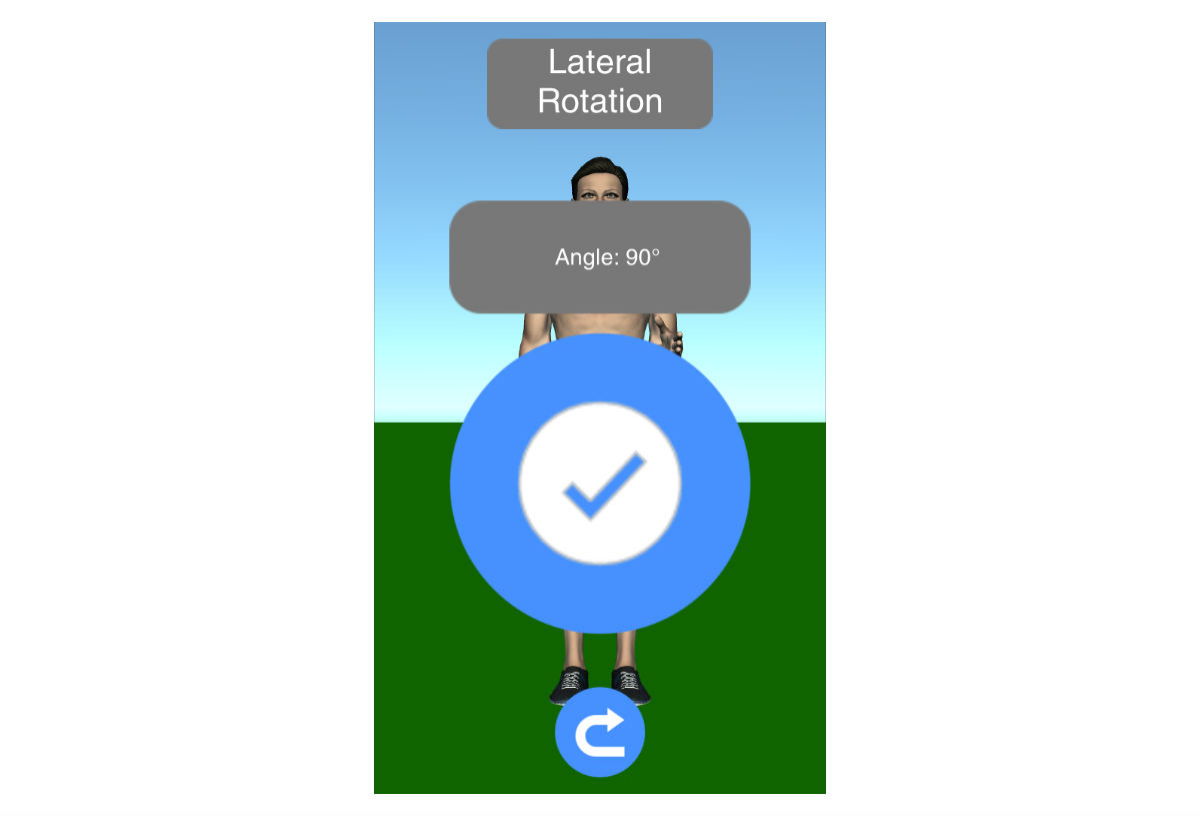
Result screen of sensor-based mobility assessment.

**Figure 7:**
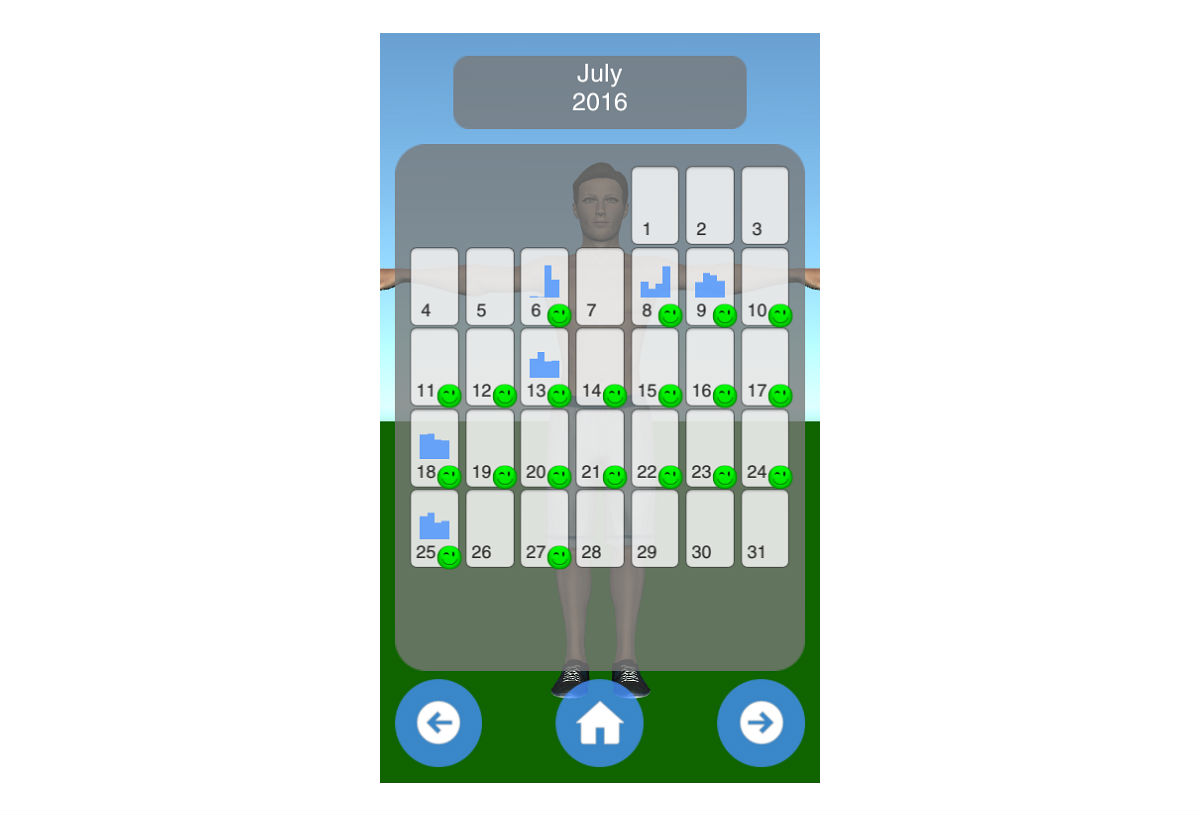
Overview screen.

Mobility assessment is useful for monitoring the progress of the effect of the treatment of frozen shoulder. For mobility assessment, two options were implemented, which can be freely chosen by the patient for each mobility assessment (see Figure 3). One mode employs manual input of the range of motion with a slider, whereas the other employs the built-in sensors of modern mobile phones. Mobility is assessed in four ways: lateral arm lift, frontal arm lift, lateral external rotation, and back rotation/scratch (see Figure 4). For sensor-based measurements, the patient uses a wrist band to attach the mobile phone to the upper arm for the lateral and frontal arm lift and on the forearm to the lateral external and back rotation. Then, the user presses the “measurement” button in an arbitrary position (see Figure 5). The patient moves the arm in a neutral (hanging) position. After 3 seconds, the measurement starts (as indicated by an audible beep) and the patient moves the arm as far as possible without any pain in the measured plane. The measurement is automatically stopped if the user moves back to the initial position. The maximum angle to the neutral position is automatically computed without any user input. After reaching the maximum position, the patient can move his or her arm into any comfortable position and examine the measurement, which is also illustrated on the avatar (see Figure 6). The patient can always repeat the measurement by pressing the “retry” button. By pressing the “ok” button, the measurement is saved. The recommendation was to conduct mobility measurement once a week. The overview screen shows a monthly calendar with a progress overview. A smiley on a day indicates that the training was carried out. Measurement results are visualized as bar charts in percent of maximum possible range of motion. Figure 7 shows the results of an overview screen of a patient included in the pilot study.

The information screen gives a brief definition of frozen shoulder and mentions the common treatment options, pain medication, and mobilization exercises. Furthermore, the most important functions of the app are briefly explained and contact information for the physician who supervised the study is given.

### Study Design

The study was designed to gather data on the feasibility of a mobile phone–based mHealth intervention for frozen shoulder. Therefore, the main focus was on usability of the app and the technology acceptance of the patients. Good usability and high technology acceptance were required for the feasibility of the intervention. Due to the limited number of available patients, a quasi-experimental design with no control group was chosen. Since the actual usage of the app at home was most relevant for the feasibility of the intervention, an ambulatory assessment of the app usage was included and app usage data was collected. In addition to usability, technology acceptance, and app usage analysis, we included an assessment of correctness of the exercise conduct. Several other outcome measures were evaluated as well (such as pain) to provide a context for the interpretation of the results and to gain an insight into their applicability in future studies.

The study design did not alter the standard physiotherapy for frozen shoulder (given as therapy order by the medical doctor). In the study, the app was employed to assist patients at home to conduct their exercises, comparable to an improved paper pamphlet. Thus, a formal approval of the federal ethics committee was not required by Austrian law. The study complied with the declaration of Helsinki [21], with the exemption of §35, which states that the study must be registered in a public database before the recruitment of the first subject.

### Participants

Inclusion criteria were a diagnosis of “stage two” frozen shoulder (frozen stage) and the willingness to voluntarily participate in the study. The participants were recruited by the physician and shoulder surgery expert, NM.

### Study Flow

The study duration was 3 weeks. Patients gave informed consent by signing a patient information sheet, including study goals and details, the voluntary participation, the data collected by the app, and a privacy statement, which informed patients that only pseudonymous information was collected during the study.

The study started with a personal meeting of each patient with a physiotherapist and a computer scientist. The exercises were explained by a physiotherapist and the app usage by a computer scientist. The patients were provided with the app either on their own phone or on a mobile phone that was provided to them. Three Android mobile phones with the preinstalled app were prepared. It was expected that the app could be installed on at least 2 patient mobile phones. Pain and movement impairments were assessed by the physiotherapist.

The patients were instructed to use the app daily to log the training, and to conduct at least one mobility assessment per week. Training and measurements were done at home and without guidance of the physiotherapist. Patients were instructed to stop training and mobility assessment in case of pain.

After 3 weeks, a second personal meeting was scheduled. Pain and movement impairments were assessed again. In this second meeting, usability questionnaires were completed by the patients. Questions about technical aspects of the interaction with the app and the study optimization from the patients’ point of view were asked as well. All questionnaires in German and English are provided in Multimedia Appendix 2. The app usage log files were collected.

### Outcome Measures and Evaluation Criteria

The outcome measures and evaluation criteria consisted of usability and acceptance questionnaires for the app, additional questions on the technical aspects of the intervention and the app, and an assessment of the correctness of the exercises, pain assessments, and mobility assessments.

#### Usability and Acceptance Evaluation

For the usability and acceptance evaluation of the app, several standardized questionnaires were employed that included selected parts (intention to use, perceived usefulness, perceived ease of use) of the revised Technology Acceptance Model (TAM-2) [22,23], the System Usability Scale (SUS) [24], and the USE (Usability, Satisfaction, and Ease of use) questionnaire, which were employed in a previous study [25]. For the interpretation of SUS scores, refer to the study by Bangor et al (2008) [26].

#### App Usage Data

The app automatically collected usage data, namely the time and date when the app was started and ended, the time and date and interaction type with the app (button push), and the results of the mobility measurement.

The duration of a single training set (20 repetitions of single exercise) were computed on the basis of these data.

#### Technical Aspects of the Intervention and the App

Furthermore, each patient was asked what they liked and what they disliked about the app. Questions on technical aspects of user interactions were asked as well, that is, whether they viewed the exercise from different angles and distances, whether they read the instructions, and whether they listened to the audio instructions. These questions were contrived by the human-computer interaction (HCI) expert and tested for understandability by the other authors.

Questions on further improvements in the overall conduct of the study and whether the initial personal instructions about how to use the app were necessary were asked.

#### Assessment of Correctness of the Exercises

In the second meeting, the physiotherapist reassessed the correctness of the exercises. The patients performed the conducted exercises under supervision of a single physiotherapist, and correctness was rated on a scale of 1 to 5:

1. No recollection of the exercise
2. Major errors, no effect of the exercise can be expected
3. Errors, effect of exercise limited
4. Minor errors, effect of exercise as presumed
5. Perfect execution

#### Assessment of Perceived Pain

The perceived pain on the first meeting (introduction of the exercises and the app) and the second meeting (interviews and evaluations) was recorded. The pain was recorded on a numeric rating scale (NRS), where 0 indicated no pain and 10 indicated the highest level of pain. Minimum pain levels (Did you experience even pain free episodes in the last days?), maximum pain (What was the worst pain you had in the last days?), and current pain levels at the time of the interview were assessed (What is your level of pain right now?). The occurrence of nightly pain was recorded as well.

#### Assessment of Mobility

In addition to the mobility assessment in the app, the ability to perform two movement tasks was assessed qualitatively by one physiotherapist at the start and at the end of the study:

1. Movement of the arm to the neck
2. Movement of the arm to the lower back

The physiotherapist explained and demonstrated the movement and recorded the ability of the patient to perform the task (“Able,” “Hardly able,” “Unable”).

## Results

In the following, the results of a 3-week pilot study with 5 patients affected by frozen shoulder are presented. The raw data are provided in Multimedia Appendices 3 – 5. The R scripts used for analysis are contained in Multimedia Appendix 6.

### Demographics and Patient Characteristics

The pilot study included 5 patients: 4 female patients and 1 male patient. The app was installed on his or her mobile phone. All patients were diagnosed with “stage two” frozen shoulder. An overview of their baseline characteristics is given in Table 1. The frozen shoulder affected the left shoulder in 3 patients and the right shoulder in 2 patients. Four patients were already treated with physiotherapy at the time of the first meeting. The patients’ participation was voluntary.

Four of the 5 patients were mobile phone users; one patient did not own and use a mobile phone but was aided by the partner, who did own a mobile phone. The partner was present in the first and second meeting and was included in the usability results, as they used the app together. Two of the 5 patients were iPhone users. Four of the 5 patients stated that they used the mobile phone for calls and text messages. Four of the 5 patients stated that they used the mobile phone for social media or messaging services. One of 5 patients also used the mobile phone for Web surfing, other apps, and health apps.

**Table 1:** Patient baseline characteristics (sorted).

Age, years	Diagnosis	Shoulder
48	February, 2014	Left
49	October, 2014	Left
56	April, 2015	Left
57	November, 2015	Right
58	November, 2015	Right

### Unexpected Events During the Study

At the first meeting, patients were provided with the app, and 3 mobile phones with the app preinstalled were prepared for users who did not have a suitable Android mobile phone. We expected that at least 2 patients owned a suitable mobile phone. However, the app could not be installed on 2 Android devices, as the devices were not satisfying the minimum system requirements (enough free space and a suitable graphic hardware). Furthermore, 2 patients were iPhone users and only a version of the app for Android at the time was provided. Thus, one patient could not use the app directly starting from the first meeting. This patient started app usage later and the study duration was only 10 days for this patient. These data are included in the analysis.

### Study Findings and Outcome Data

In the following, the results on the changes of perceived pain, app usage, and compliance; the correctness of the exercise conduct; technical aspects of the app; and usability questionnaires are presented.

#### Usability Questionnaires

The results of the usability questionnaires are summarized in Table 2. TAM-2 answers were given on 5-point Likert scale, from 1 (negative/disagree) to 5 (positive/strongly agree). The TAM-2 results are summarized in Table 2. The users (5 patients and the partner of 1 patient) showed strong intention to further use the app (4.2 on an average); only one patient reported that she/he did not like regular usage of mobile phones at all and she/he would not like to use such apps. The users considered the app useful. The average score for perceived usefulness was 3.9. The users considered the app easy to use. The average score for perceived ease of use was 4.3.

The questions of the USE questionnaire were rated on a 5-level Likert scale as well. The users considered the app easy to learn. The average score for ease of learning was 4.2. The users were satisfied with the app. The average score for satisfaction was 4.7.

The app achieved an average SUS score of 88 (on a 0 to 100 scale), which indicates a very usable system [26].

**Table 2:** Results of the usability questionnaires (n=6). Technology Acceptance Model-2 (TAM-2) and Usefulness, Satisfaction, and Ease of use (USE) scores range from 1 to 5 (best score). System Usability Scale (SUS) ranges from 0 to 100 (best score). SUS score above 80 indicate highly usable systems.

Questionnaire	Mean	Standard deviation
TAM-2^a^: Intention to use	4.2	1.5
TAM-2: Perceived Usefulness	3.9	0.8
TAM-2: Perceived Ease of Use	4.4	0.5
USE^b^: Ease of Learning	4.2	0.8
USE: Satisfaction	4.7	0.8
SUS^c^	88	6

^a^TAM-2: Technology Acceptance Model-2.

^b^USE: Usefulness, Satisfaction, and Ease of use.

^c^SUS: System Usability Scale.

#### Compliance and Quantitative Usage Data

**Figure 8:**
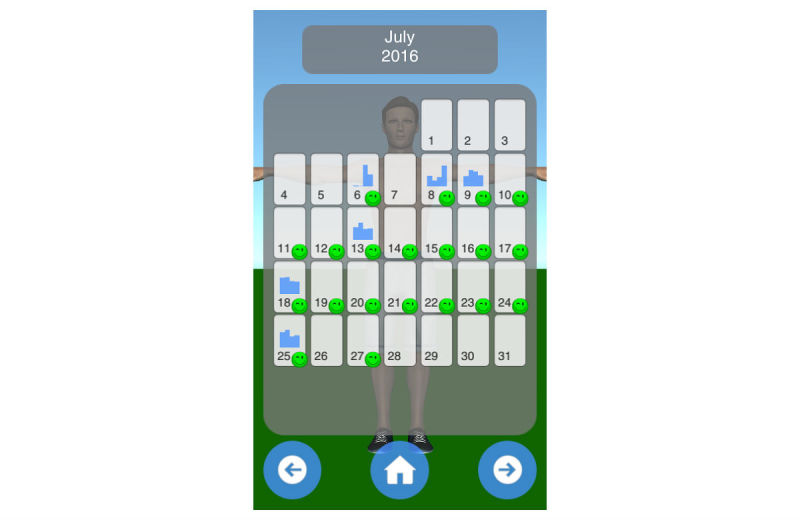
Overview screen of patient with PID 01.

**Figure 9:**
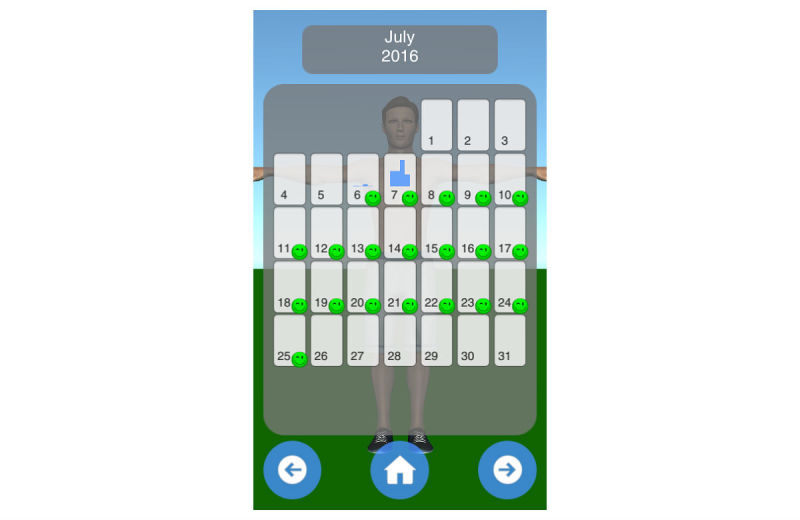
Overview screen of patient with PID 02.

**Figure 10:**
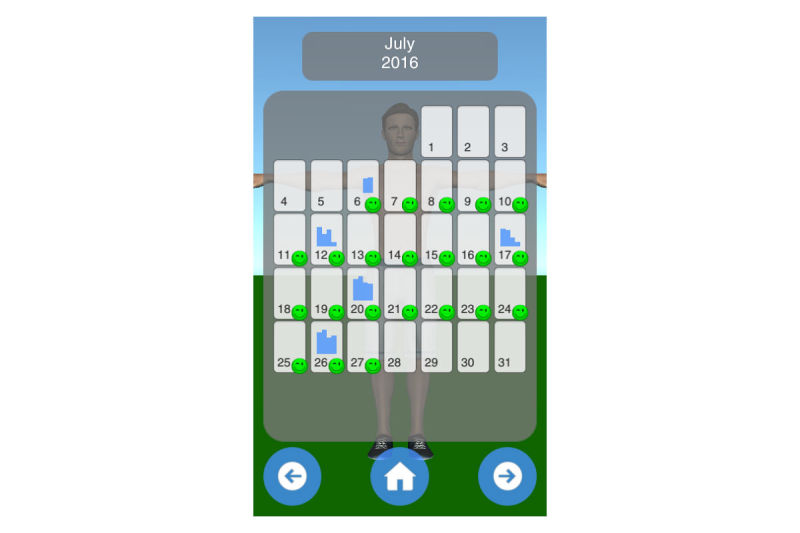
Overview screen of patient with PID 03.

**Figure 11:**
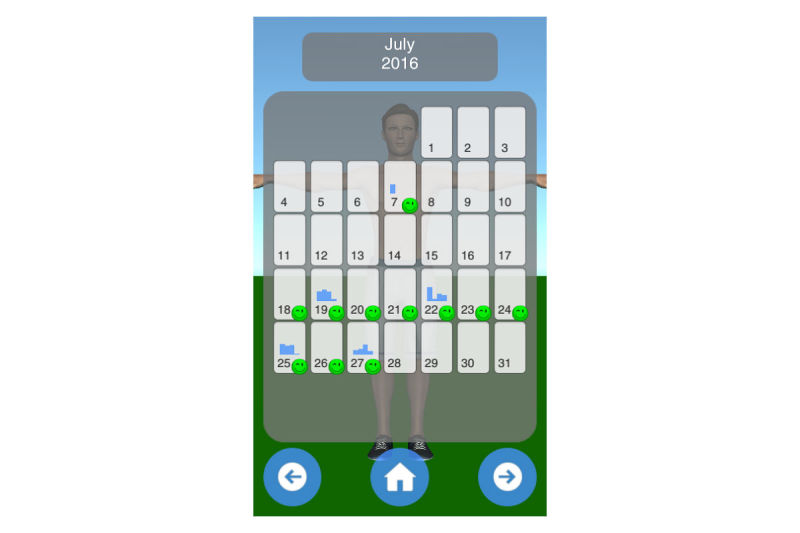
Overview screen of patient with PID 04.

**Figure 12:**
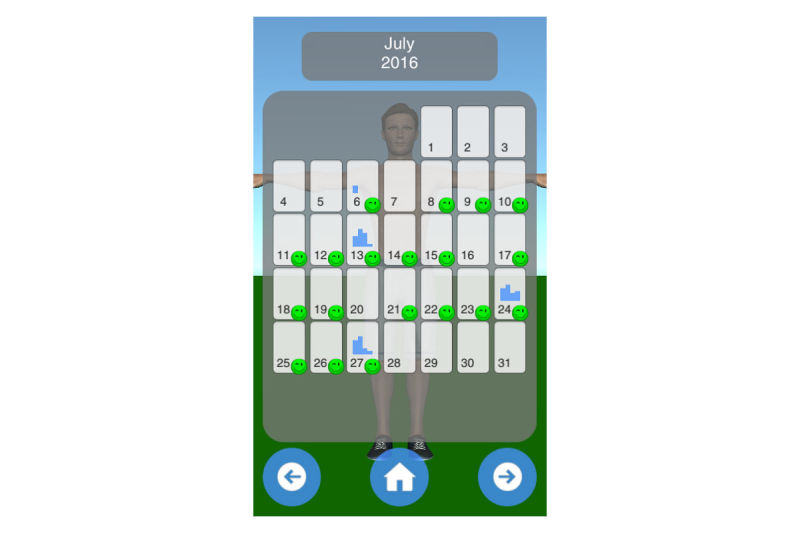
Overview screen of patient with PID 05.

All patients reported that they used the app. The patient statements were verified by the log files of the app; the overview screens of the patients are shown in Figures 8-12. A green smiley refers to a training session. A blue bar plot represents the result of a mobility assessment (the higher the bar, the more mobile the patient’s shoulder joint). The patients performed the training on every day of the study (green smileys), but one patient started later (see Figure 11). All patients except one assessed their mobility at least once a week during the study (a bar plot in the Calendar represents a mobility assessment).

**Figure 13:**
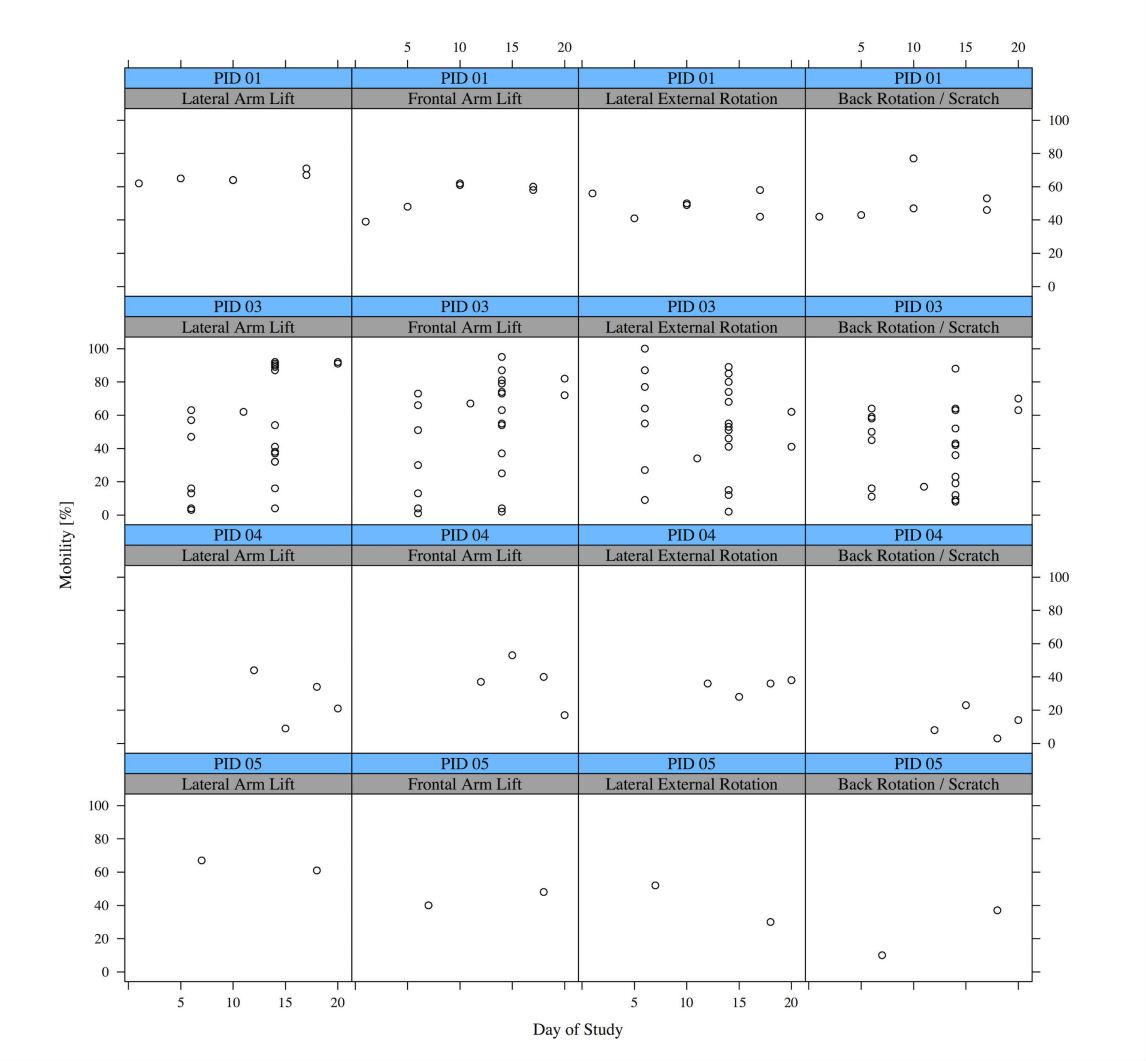
All mobility measurements for each patient and all four mobility assessments (n=4, N=139). Each square contains the measurements for a certain patient and a certain assessment method, that is, Lateral Arm Lift. The mobility measurement is given in percent of the maximum possible mobility range and plotted against the day of study.

In the further analysis of the quantitative usage data, the first day (instruction day) and the last day (end of study) were excluded in order to omit the instruction and reporting usage cases of the app. Especially interesting is a closer investigation of the mobility measurements with the app. Figure 13 shows all measurement results per patient; each circle visualizes one distinct mobility measurement. We excluded one patient (PID 02), who did not record any mobility measurements after the first meeting. One patient (PID 03) repeated the mobility measurements multiple times until she/he was satisfied. Overall, 139 single mobility assessments were successfully completed, 32 mobility assessments were interrupted (eg, by pressing the “home” button, an incoming call), and for 21 mobility assessments, the slider was not touched at all.

Another interesting question is how the training mode of the app was used. Namely, did the patients just quickly mark the exercises as done, or did they use the training mode to guide them through the exercises?

The avatar executes a single repetition of an exercise within 3.5 seconds, that is, 70 seconds for a set of 20 repetitions, and the time for the relaxation phase between sets was not specified. The minimum plausible time for a set when using the app during exercising was 20 seconds, as a single repetition of one exercise requires at least one second based on practical tests by the research team. The maximum plausible time for using the app during exercising was set to 200 seconds, that is, about 3 minutes for 20 repetitions and a relaxation phase.

**Figure 14:**
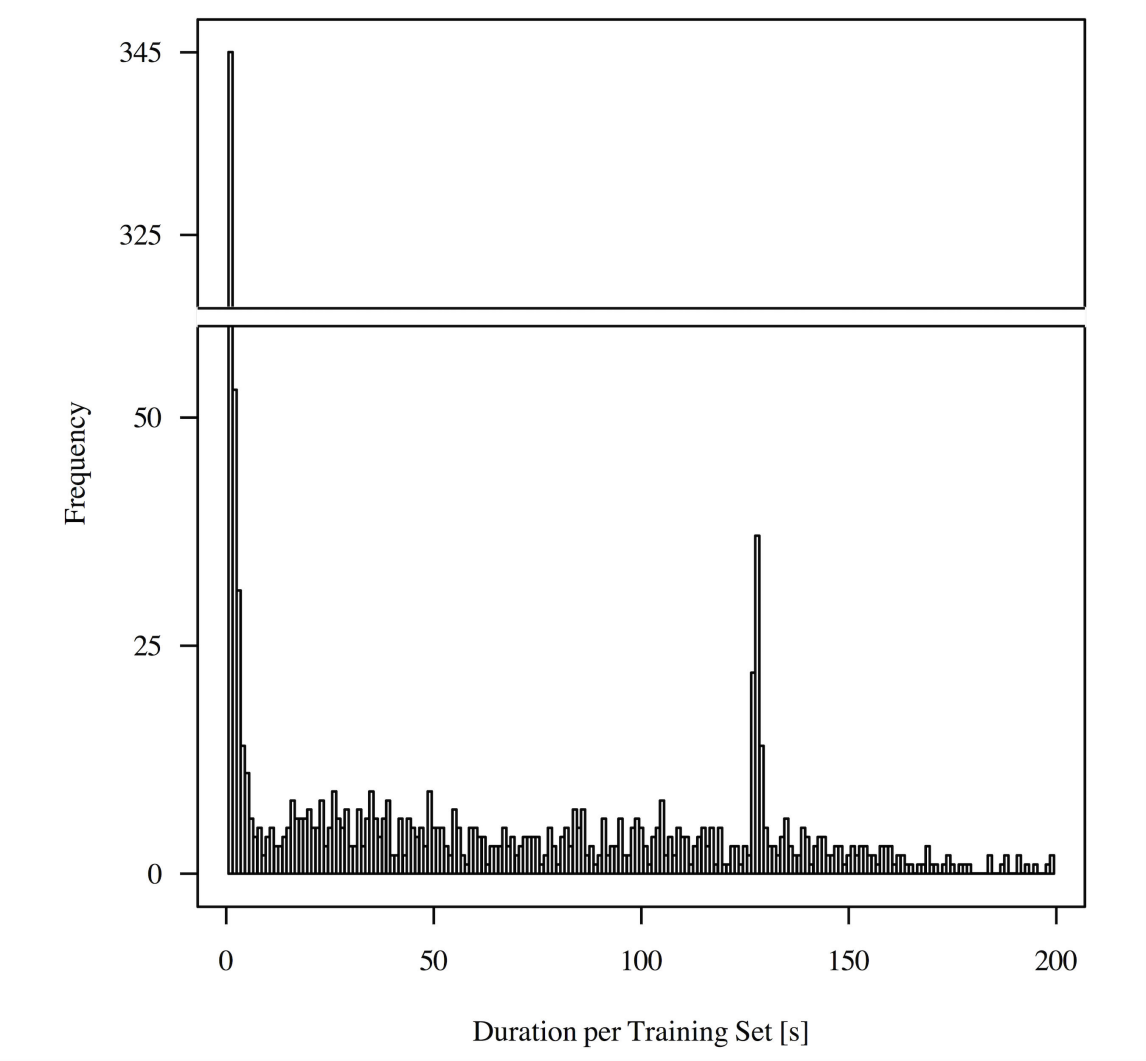
Histogram of training set durations (n=5, N=1145).

Our analysis shows that for more than half of the time (624 out of 1145), the patients used the app during training and did not just tick off the exercises. Figure 14 shows a histogram of the duration of a single set of an exercise. Many durations of a single set are close to zero; in these cases, the patients used the app often just to tick off exercises. A smaller peak at 125 seconds can be observed, which corresponds to the recommended set time (70 seconds) and less than a minute of relaxation between the sets.

The patients were instructed to conduct the four exercises with three sets each on a daily basis (4 patients for 20 study days and 1 patient for 9 study days), that is, for perfect compliance 1068 exercise sets were expected. As 1260 exercise sets were recorded, training compliance was excellent. Of these 1260 exercise sets, 78 sets had durations of above 200 seconds and were therefore excluded. 37 exercise sets were interrupted (eg, by pressing the “home” button, turning off the phone, or receiving a call) and were therefore excluded as well.

Four hundred and sixty sets had durations shorter than 7 seconds, that is, in these cases it was concluded that the app was only used to mark the exercises as completed. In addition, 61 set-durations were too long for just checking the exercise sets as done, and too short to properly conduct the exercise set. An explanation could be that the patients showed the exercises to someone.

**Figure 15:**
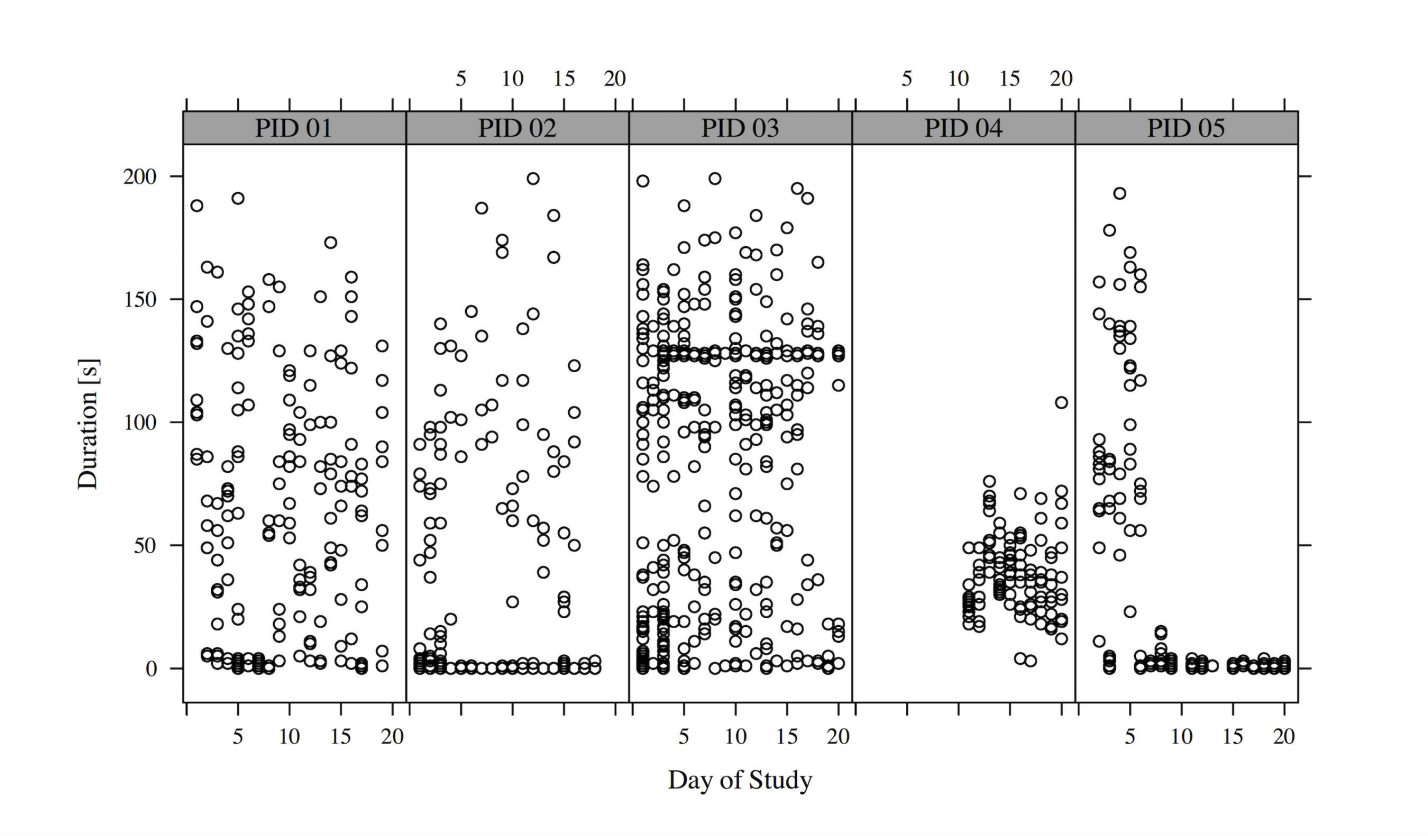
Training set durations per patient (N=1145).

Figure 15 shows the set duration per patient and day of study. One patient (PID 05) stopped to use the app during training, and started to use the app only for confirmation after a week.

**Figure 16:**
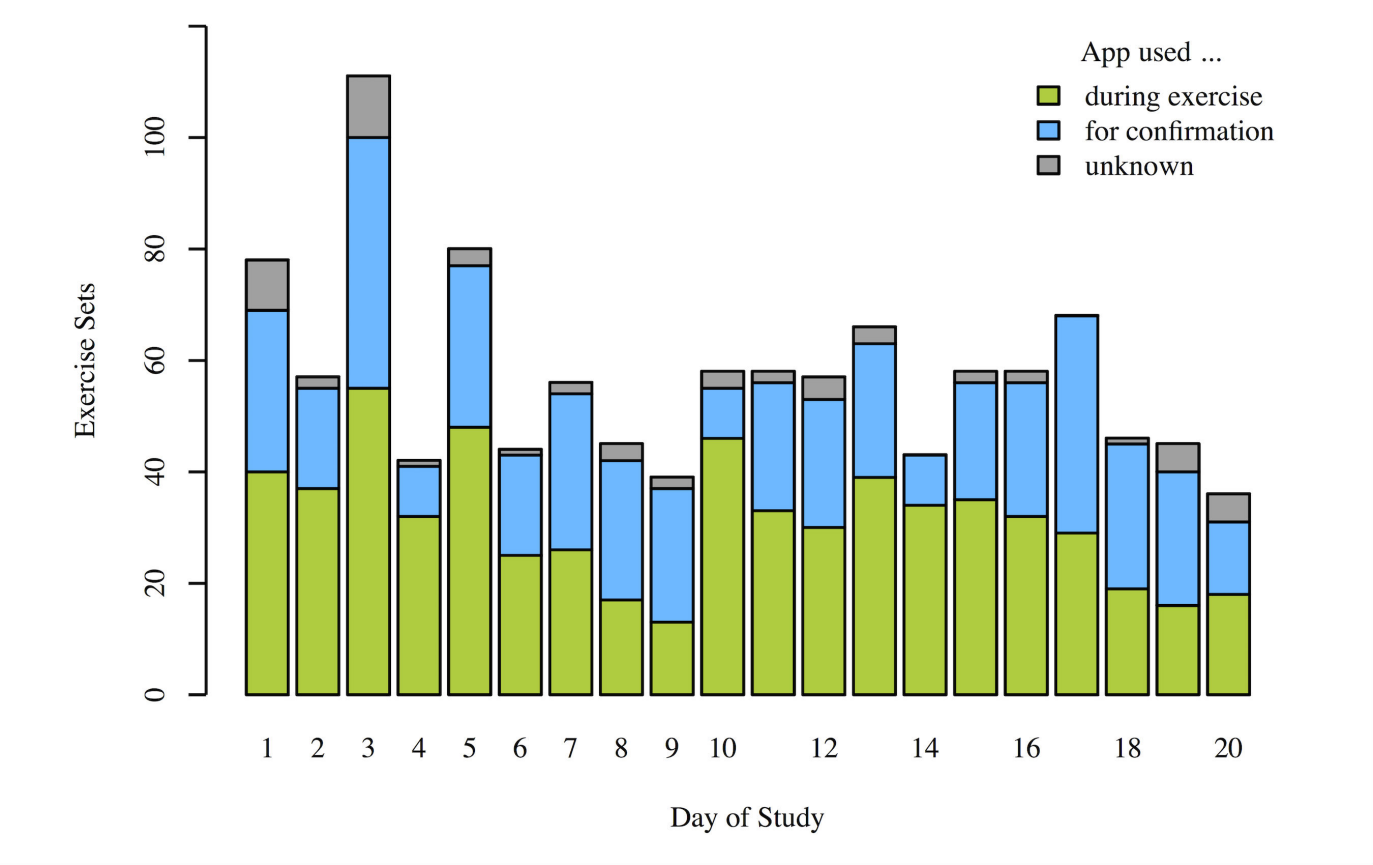
Training mode usage pattern per study day (n=5, N=1145).

Figure 16 shows the usage patterns of the app over time. Green bars illustrate the sets that have likely been completed using the app during the exercise (set durations between 20 and 200 seconds), whereas blue bars illustrate the percentage of sets that used the app just to tick off the exercises (confirmation, set durations below 7 seconds). Gray bars (label “unknown”) refer to set durations above 7 seconds and below 19 seconds. Overall, compliance stayed high during the study. For perfect compliance, each patient had to perform 12 exercise sets per day, that is, 48 exercises for the 4 patients of the first 11 days of the study and 60 exercise sets for the 5 patients for the rest of the study.

#### Technical Aspects of the Intervention and the App

In the following, the feedback to each question about technical aspects of the app is summarized. In order to preserve the privacy of the patients, all information is given as generally as possible.

##### Q1: What Did You Like About the App and the Intervention?

One patient said that he/she liked that the app motivated her to regularly and properly conduct the exercises. One patient reported that he/she especially liked a certain exercise (stretching in the door). The partner of one patient reported that they conducted the manual mobility assessment (without sensors) together and that the joint usage of the app was enjoyable. One patient reported that she/he liked the simplicity of the program and that the app would even be usable for someone with no mobile phone usage experience. The introduction to the app and the exercises in the first meeting were mentioned as well. Furthermore, the possibility to contact the physician during the trial was positively noted.

##### Q2: What Did You Not Like About the App and the Intervention?

Two patients and the partner of one patient reported that there was nothing they did not like. One patient deemed the instructions for the mobility assessment as insufficient. One patient said that despite owning a mobile phone, she/he does not like to use it and does not like to report on a daily basis. One patient reported that a different choice of wrist bands should be considered, as due to the design of the distributed wrist bands, these had to be tediously adjusted for the measurements on the upper and the lower arm. One patient said that the current manual mobility assessment required a second person.

##### Q3: Did You Change the Viewpoint of the Avatar?

All the patients and the partner reported that they changed the view point, in order to view the exercises from different angles and to have better control of their own conduct of the exercise.

##### Q4: Did You Read the Exercise Text Instructions?

Only 2 patients reported that they did not read the instructions at all; 3 patients and the partner of one patient used the text instructions.

##### Q5: Did the Audio Explanation of the Exercises Help?

Three patients said the audio was helpful. Two patients and the partner of one patient did not find the audio instructions helpful.

##### Q6: Did You Use the Mobility Assessment With the Mobile Phone Sensors?

Three patients used the assessment with the sensors. One did not know how to conduct the measurements and one mobile phone did not support the sensor measurement. Furthermore, one patient slightly misunderstood the measurement process, which made the measurement process more cumbersome, as she/he thought she/he had to press the “accept measurement” button at the maximum angle of movement.

##### Q7: Would You Like to Document the Pain With the Mobility Assessment?

Three patients and the partner of one patient did not like to document pain. Two patients would have liked to document the pain, but did not have a suggestion on how they would like to do it.

##### Q8: What Could the Study Organizers Have Done Better?

Two patients reported that the sensor-based mobility assessment would benefit from better instructions in the first meeting and in the app. One patient recommended that at least one measurement should be done by the patient in the first meeting. Furthermore, one patient suggested more exercises (also for back pain) and a selection of exercises more specifically chosen to the individual patients’ condition and impairment.

##### Q9: Did You Need the Personal Instructions for the App?

Three patients and the partner reported that they needed the instructions. One patient said that only the mobility assessment needs instructions and that patients should be encouraged to perform a self-measurement during the initial instructions. One patient said that the personal instructions were not necessary.

#### Assessment of Correctness of Exercises

All the patients reported that no improvements of the exercise instructions were necessary. All of them thought that they conducted the exercises correctly (4, 4, 5, 5, 5; with 1 having no memory how to perform the exercise and 5 being totally correct). Four of the 5 patients could participate in the second meeting in person; one patient was ill and was interviewed by telephone. Thus, the correct conduct was only assessed for 4 patients. The assessment of the physiotherapist confirmed the correctness of the conduct of the exercises. Only minor differences to the optimal exercise conduct were present (see Table 3 for detailed comments).

**Table 3:** Assessment of the correctness of exercises.

Patient	E1^a^	Comment	E2	Comment	E3	Comment	E4	Comment
PID 01	4	Seat to high	4	Seat to high	5		4	Elbow not bent enough
PID 02	5		4	Upper body slightly too upright	4	Legs not bent	4	Elbow bent too much
PID 03	4	Sometimes small circular movements	5		4	Legs not bent	5	
PID 04	5		5		4	Legs not bent	5	

^a^The exercises 1 to 4 (E1 to E4 in the heading) were assessed by a physiotherapist on a scale of 1 (no recollection) to 5 (perfect execution).

#### Pain and Mobility Assessments

The results for the grades of pain are summarized in Table 4. Decreased pain levels are colored in green; increased pain levels are colored in red and with a horizontal stripe pattern. Minimum pain levels (NRS min in Table 4) increased slightly for Patient PID 02 (from 0 to 1.5). Patient PID 05 had a decrease in minimum pain from 3.5 to 2. Maximum pain levels (NRS max in Table 4) were reduced in 4 patients (decreased by 2.5, 0.5, 1 and 0.5, respectively) and increased by 0.5 in patient PID 05. All patients reported reduced current pain levels. Pain during the night remained constant for all patients (three were affected by nightly pain, two did not).

Additionally, two movement tasks were tested, namely moving the hand to the neck and moving the hand to the lower back (Table 5). For one patient, an improvement for the first movement was recorded (from hardly possible to possible), and for another patient, an improvement for the second movement was noticeable (from not possible to hardly possible).

**Table 4:** Grades of pain at the start and the end of the study in a numeric rating scale (NRS).

Patient	NRS min^a^		NRS max^b^		NRS current^c^			Nightly pain	
	Start	End	Start	End	Start	End	Start		End
PID 01	0	0	5	2.5	1	0	No		No
PID 02	0	1.5	9	8.5	2.5	1	Yes		Yes
PID 03	0	0	2.5	1.5	1.5	0	Yes		Yes
PID 04	0	0	2.5	2	0	0	No		No
PID 05	3.5	2	3.5	4	3.5	2	Yes		Yes

^a^NRS values range from 0 (no pain) to 10 (high pain). NRS min refers to the minimum perceived pain in the last days.

^b^NRS max refers to the maximum perceived pain in the last days.

^c^NRS current refers to the pain level during the interview.

**Table 5:** Performance on movement tasks at the start and the end of the study.

Patient	Task 1		Task 2	
	Start	End	Start	End
PID 01	Able	Able	Hardly able	Hardly able
PID 02	Able	Able	Unable	Able
PID 03	Able	Able	Hardly able	Hardly able
PID 04	Hardly able	Able	Able	Able
PID 05	Able	Able	Hardly able	Hardly able

### Unexpected Observations

Two patients reported joint usage of the app with their partner. One patient was no mobile phone user, and used the app together with the partner on the partner’s device. One patient reported that the partner assisted in the mobility assessment.

## Discussion

### Answer to the Study Questions

The main research question of this work was whether the mobile phone app-based mHealth intervention is feasible. Considering the satisfying results in the usability evaluation and the fact that the patients actually used the app at home and could correctly perform the exercises, a strong case for the feasibility of the mHealth intervention can be made. On the basis of the analysis of the quantitative app usage data, the conclusion is drawn that excellent compliance was achieved for both training mode and the assessment of mobility. The designed app was shown to be a suitable support tool that was accepted by the majority of the small study population. The exercise instructions worked well and the 3D interaction was a beneficial and frequently used feature. The problem of uncertainty regarding how to perform an exercise (a common reason to avoid exercising [27]) was solved for the selected frozen shoulder exercises.

Overall, the app tackled important obstacles for physiotherapy at home via comprehensible and easily accessible exercise instructions, compliance, exercise correctness, and progress monitoring [28,29].

### Strengths and Weaknesses of the Study

Our usability evaluation was based on a 3-week ambulatory assessment with real patients using the app at their real home and not in a controlled laboratory setting, which can raise many issues that are not illuminated in a lab or hypothetical setting [30]. Therefore, we believe that our evaluation and system are close to the actual requirements of home-based physiotherapy [28,29]. However, only 5 patients took part in the pilot study and a certain positive bias might have been introduced by the study design.

### Results in Relation to Prior Work

There has been a significant interest of the research community and the industry in technology assistance for rehabilitation and health and fitness.

Apart from general health and fitness, which have become topics for major companies such as Google (Google Fit) and Apple (Apple Health), several specific medical and rehabilitation issues have been addressed in the HCI and the medical community. Among these issues were stroke rehabilitation [31], Parkinson disease [32], cerebral palsy [33], autism [34], and most importantly, for the focus of this study, musculoskeletal disorders (MSDs) [28,29], including the disorders of the knee [35] and the shoulder [36,37].

Previous studies on technology assistance for rehabilitation and health and fitness can be classified in terms of the used technology and hardware, which range from the application of professional tracking hardware [38] over virtual and augmented reality HMDs (head mounted displays) [39] and mainstream gaming hardware [40] to everyday mobile phones [41-43].

#### Non-Mobile Phone–Based Systems

Professional tracking systems are capable of precisely tracking patient motion during exercises and use these data to provide feedback. A Vicon tracking system was used to implement a prototype for physiotherapy at home [44,38].

Virtual reality (VR) HMDs offer the efficient simulation of training environments. VR systems were used to simulate situations of everyday life (eg, a virtual kitchen) where patients with cognitive disabilities could relearn daily living skills [45]. VR exer-game, in which the user controls the avatar movement with an ergometer, was proposed as well [46]. However, as compared with a mobile phone app, a VR system is not as suitable for home exercising and wide deployment, as it requires expensive hardware to be installed at the home of the patient.

Augmented reality (AR) systems with HMDs (such as the Microsoft HoloLens) allow to graphically overlay the visual perception with additional information, which would be well-suited to provide patients with feedback on exercise performance. The design of AR games for upper extremity motor dysfunctions was investigated [47] and in a follow-up study, an AR game for an HMD system was evaluated [39]. However, as compared with a mobile phone, AR HMDs are expensive and not widely available at the moment.

Off-the-shelf game console hardware has been proposed to support physiotherapy. The accuracy of Microsoft’s Kinect body tracking for rehabilitation purposes was quantitatively assessed [48]. Kinect-based systems for physiotherapy have been proposed [40,49]. A Kinect-based system for shoulder impingement therapy was presented as well [36]. The Nintendo Wii system includes a game controller that allows pointing at screen positions and contains an accelerometer. Rehabilitation of cerebral palsy with a system running on the Nintendo Wii was investigated [33]. Off-the-shelf Nintendo Wii Fit games were employed and evaluated with respect to the retention of motor skills of patients with Parkinson disease [32].

However, as compared with mobile phones, even gaming consoles are not as widely deployed, especially for individuals in the age group of 40 to70 years. Furthermore, the small movements of the exercises for frozen shoulder are hard to track with off-the-shelf hardware. Even recordings of our exercises with a professional motion capturing system (OptiTrack) required manual corrections by a 3D animator.

Accelerometers and gyroscopes, that is, inertial measurement units (IMUs), have been widely used in previous studies on technology-assisted rehabilitation. An IMU-sensor–based system to deliver balance and strength exercises to the elderly was proposed [50]. Knee rehabilitation supported by IMUs was proposed [35,51]. A cap with an IMU (Sense-Cap) to monitor balance exercises was proposed and evaluated [30]. A more complex IMU-based system to provide motion guidance was also proposed [52]. Compared with our system, additional hardware (IMUs) needs to be distributed to the patients.

#### Mobile Phone–Based Systems

Mobile phone apps for general health and fitness have moved from research to practice. The application of mobile phone apps in medical and rehabilitation contexts is currently heavily researched.

Early studies [41,53] proposed a context-aware and user-adaptive mobile system for fitness training. A 3D avatar was used as a mobile trainer and to show the exercises. It was pointed out that the use of a 3D avatar allowed the user to perform the exercises more accurately.

The use of conversational interfaces for health and fitness companions was discussed [54]. User-tailored activity coaching systems were reviewed [55]. Mobile phone apps were investigated for physiotherapy [43]. A reminder app for stroke patients was proposed [56]. A mobile phone app to encourage activity in patients with chronic obstructive pulmonary disease was evaluated [25].

There are a large number of commercial fitness and training apps. In these apps, exercises are presented using animated videos (no view point change is possible). None of the commercially available mobile phone applications use an interactive 3D avatar, which our system offers.

Physiotherapy over video communication was discussed and evaluated [57]. It was highlighted that information of bodily cues is limited in two-dimensional videos.

Compared with most of the previous contributions from academia, which have mainly focused on special not widely available hardware (especially in the age group of 40 to 70), our proposal only requires a standard mobile phone.

Previous studies show, that new technology is hardly accepted by many elderly patients [58] and especially, hardware that has to be installed at home is problematic [31].

Although our app is not the first app to target MSDs, it is the first that specifically tackles frozen shoulder and presents an evaluation on the basis of a pilot study.

### Meaning and Generalizability of the Study

Treatment options of frozen shoulder have not been assessed conclusively so far, and our contribution cannot provide this assessment. However, our results indicate that the frozen shoulder app can play an important role in patient motivation, exercise instruction, and shoulder mobility progress assessment. Therefore, the frozen shoulder app may also be employed in the evaluation process of other treatment options for frozen shoulder (mobility monitoring). The presented app can be considered the first part of a system for a thorough and standardized evaluation of home-exercise–based physiotherapy for frozen shoulder. Such a system can support the assembly of high quality evidence for the treatment options of frozen shoulder.

### New Questions and Future Research/Improvements

Overall, the positive patient feedback and the results justify further work on the app to support the treatment of frozen shoulder. In the course of the study, the physiotherapists proposed the integration of a training’s planning mode, which offers more exercises and the adaptation of the number of sets and the iterations per set. The training’s planning mode also enables to adapt the app more to the specific requirements of a single patient. Furthermore, physiotherapists proposed to include the possibility to add personalized information for the patient (text, audio, video). As 2 patients reported joint usage of the app with their partner, the further integration of the social contacts (partners, friends) in the app usage and training could be investigated.

Our analysis also highlights that instructions for the mobility measurement need to be improved and the repeatability and reliability of the self-measurement process of the patients need to be carefully investigated. Users with no mobility limitations achieved almost perfect repeatability of the measurements. Given that in over 50% of the exercise sets the app was used while training but the set durations varied greatly, the inclusion of explicit timing information (a counter) should be considered.

### Conclusions

A mobile phone app to support the therapy of patients with frozen shoulder was developed. Overall, the proposed mobile phone–based mHealth intervention was shown to be feasible. Main obstacles of home-based physiotherapy could be tackled, as the mobile phone-supported intervention resulted in correct exercise conduct and high compliance. The patients reported high technology acceptance and very good usability.

## Appendix

Multimedia Appendix 1 FrozenShoulder app.

Multimedia Appendix 2 All questionnaires (original German and English translation).

Multimedia Appendix 3 Excel-sheets of results of all questionnaires.

Multimedia Appendix 4 Application log file.

Multimedia Appendix 5 Application log file.

Multimedia Appendix 6 Evaluation scripts for log files.

## Abbreviations

AR: augmented reality
HCI: human-computer interaction
HMD: head mounted displays
ICD: International Classification of Diseases
IMU: inertial measurement units
mHealth: mobile health
MSD: musculoskeletal disorders
NRS: numeric rating scale
SUS: System Usability Scale
TAM-2: Technology Acceptance Model-2
3D: three-dimensional
USE: Usefulness, Satisfaction, and Ease of use
VR: virtual reality
