# Supplementary material for: Mobile Phone–Supported Physiotherapy for Frozen Shoulder: Feasibility Assessment Based on a Usability Study
Source: JMIR Rehabil Assist Technol. 2017 Jul 20;4(2):e6. doi: 10.2196/rehab.7085 (PMC5544899; doi:10.2196/rehab.7085)
Supplement: Multimedia Appendix 2 [file rehab_v4i2e6_app2.pdf]

PatientInnen-Interview zur Prestudy Frozen Shoulder

Beschreibung der Stichprobe

Alter:
Geschlecht:
Krankheitsbeginn:
Andere chronische Krankheiten:

Smartphone-Nutzungsverhalten
Besitzen Sie ein Smartphone?
Ja / Nein
Falls ja: Nutzen Sie Ihr Smartphone

- zum Telefonieren und SMS,
- für Soziale Dienste und Kurznachrichten (z.B., Facebook, WhatsApp, Skype)
- Internet-Surfen
- für andere Apps
- für Fitness / Health-Apps)

Quality Of Life / Coping

Wie ist es Ihnen in den letzten drei Wochen gegangen?

Haben Sie die App verwendet?
Ja / Nein

➔ Falls Nein: Warum nicht?

Haben Sie das Gefühl, die App hat Sie in den letzten 3 Wochen bei Ihrer Krankheit unterstützt?

**überhaupt nicht**<br>**1**	**2**	**3**	**4**	**Sehr stark**<br>**5**
○	○	○	○	○

TAM2

Nutzungsabsicht / Intention to Use

1. Angenommen ich könnte die App verwenden, dann würde ich mir vornehmen, die App verwenden.

**Stimme überhaupt nicht zu**<br>**1**	**2**	**3**	**4**	**Stimme voll zu**<br>**5**
○	○	○	○	○

2. Angenommen ich könnte die App verwenden, dann glaube ich, dass ich die App tatsächlich verwenden würde.

**Stimme überhaupt nicht zu 1**	**2**	**3**	**4**	**Stimme voll zu 5**
○	○	○	○	○

Wahrgenommen Nützlichkeit / Perceived Usefulness

1. Die Verwendung der App hilft mir bei meiner Schultererkrankung.

**Stimme überhaupt nicht zu 1**	**2**	**3**	**4**	**Stimme voll zu 5**
○	○	○	○	○

2. Die Verwendung der App führt bei mir zu einer erhöhten Übungshäufigkeit.

**Stimme überhaupt nicht zu 1**	**2**	**3**	**4**	**Stimme voll zu 5**
○	○	○	○	○

3. Die Verwendung der App führt zu einer genaueren Übungsdurchführung.

**Stimme überhaupt nicht zu 1**	**2**	**3**	**4**	**Stimme voll zu 5**
○	○	○	○	○

4. Ich finde die App nützlich.

**Stimme überhaupt nicht zu 1**	**2**	**3**	**4**	**Stimme voll zu 5**
○	○	○	○	○

Wahrgenommen Benutzbarkeit / Perceived ease of use

1. Ich fand die Interaktion mit der App einfach und verständlich.

**Stimme überhaupt nicht zu 1**	**2**	**3**	**4**	**Stimme voll zu 5**
○	○	○	○	○

2. Die Benutzung der App erfordert nicht viel Aufmerksamkeit und Überlegen (mental effort).

**Stimme überhaupt nicht zu 1**	**2**	**3**	**4**	**Stimme voll zu 5**
○	○	○	○	○

3. Die App war für mich einfach zu benutzen.

**Stimme überhaupt nicht zu 1**	**2**	**3**	**4**	**Stimme voll zu 5**
○	○	○	○	○

4. Die App stellt die relevanten Funktionen einfach zur Verfügung.

Stimme überhaupt nicht zu 1	2	3	4	Stimme voll zu 5
◯	◯	◯	◯	◯

Technische Aspekte:

Was hat Ihnen besonders gut gefallen?

Was hat Ihnen nicht gefallen?

Haben Sie die Kameraansicht verändert?
Ja/nein/Warum?
Haben Sie die Texte mit den Übungsanweisungen gelesen?
Ja/nein/Warum?
Wenn ja, haben Ihnen die vorgelesenen Übungsanweisungen geholfen?
Ja/nein/Warum?
Haben Sie die Beweglichkeitsmessungen mit den Sensoren verwendet?
Ja/nein/Warum?
Würden Sie gerne bei der Messung den Schmerz mitprotokollieren?
Ja/Nein
Wenn ja, wie?

Was hätten wir besser machen können? (nicht nur App, auch Einschulung, beim Kontaktieren)

Hätten Sie die Einschulung gebraucht (in die App)?
Ja / Nein

Korrektheit der Durchführung und Vermittlung

Haben Sie das Gefühl die Übungen korrekt durchgeführt zu haben?

Nein, überhaupt nicht 1	2	3	4	Sehr stark 5
◯	◯	◯	◯	◯

Wie könnten wir die Übungsvermittlung verbessern?

Können Sie uns die Übungen vorzeigen? [Qualität der Übung wird von Physiotherapeutin begutachtet, es wird auf Missverständnisse in der Vermittlung durch die App eingegangen]
Übung 1:

Nicht erinnert<br>1	Große Fehler,<br>Wirkung der Übung nicht gegeben<br><br>2	Mittlere Fehler,<br>Übungswirkung eingeschränkt<br><br>3	Minimale Fehler,<br>Übungswirkung sollte gegeben sein<br><br>4	Vollständig richtig<br>5
○	○	○	○	○

Kommentar:

Übung 2:

Nicht erinnert<br>1	Große Fehler,<br>Wirkung der Übung nicht gegeben<br><br>2	Mittlere Fehler,<br>Übungswirkung eingeschränkt<br><br>3	Minimale Fehler,<br>Übungswirkung sollte gegeben sein<br><br>4	Vollständig richtig<br>5
○	○	○	○	○

Kommentar:

Übung 3:

Nicht erinnert<br>1	Große Fehler,<br>Wirkung der Übung nicht gegeben<br><br>2	Mittlere Fehler,<br>Übungswirkung eingeschränkt<br><br>3	Minimale Fehler,<br>Übungswirkung sollte gegeben sein<br><br>4	Vollständig richtig<br>5
○	○	○	○	○

Kommentar:

Übung 4:

Nicht erinnert<br>1	Große Fehler,<br>Wirkung der Übung nicht gegeben<br><br>2	Mittlere Fehler,<br>Übungswirkung eingeschränkt<br><br>3	Minimale Fehler,<br>Übungswirkung sollte gegeben sein<br><br>4	Vollständig richtig<br>5
●	○	○	○	○

Kommentar:

USE-Fragebogen

Leichtigkeit des Erlernens der App (Ease of Learning)

Ich lernte schnell die App zu verwenden.

Stimme überhaupt nicht zu 1	2	3	4	Stimme voll zu 5
◯	◯	◯	◯	◯

Es fiel mir leicht mir die Verwendung zu merken.

Stimme überhaupt nicht zu 1	2	3	4	Stimme voll zu 5
◯	◯	◯	◯	◯

Es war leicht die Verwendung der App zu erlernen, insbesondere Messung mit den Sensoren.

Stimme überhaupt nicht zu 1	2	3	4	Stimme voll zu 5
◯	◯	◯	◯	◯

Ich wurde schnell geschickt im Umgang mit der App (insbesondere der Messung mit den Sensoren).

Stimme überhaupt nicht zu 1	2	3	4	Stimme voll zu 5
◯	◯	◯	◯	◯

Zufriedenstellung (Satisfaction)

Ich bin mit der App zufrieden.

Stimme überhaupt nicht zu 1	2	3	4	Stimme voll zu 5
◯	◯	◯	◯	◯

Ich würde die App einem/r anderen PatientIn empfehlen.

Stimme überhaupt nicht zu 1	2	3	4	Stimme voll zu 5
◯	◯	◯	◯	◯

Es macht Spaß die App zu benutzen.

Stimme überhaupt nicht zu 1	2	3	4	Stimme voll zu 5
◯	◯	◯	◯	◯

Die App funktioniert so wie ich es mir vorstelle.

Stimme überhaupt nicht zu 1	2	3	4	Stimme voll zu 5
◯	◯	◯	◯	◯

Die App ist wunderbar.

Stimme überhaupt nicht zu 1	2	3	4	Stimme voll zu 5
◯	◯	◯	◯	◯

Ich will die App haben.

Stimme überhaupt nicht zu 1	2	3	4	Stimme voll zu 5
◯	◯	◯	◯	◯

Die App ist angenehm zu benutzen.

Stimme überhaupt nicht zu 1	2	3	4	Stimme voll zu 5
◯	◯	◯	◯	◯

Dieser Fragebogen war verständlich.

Stimme überhaupt nicht zu 1	2	3	4	Stimme voll zu 5
◯	◯	◯	◯	◯

Begründung:

Dieser Fragebogen war einfach auszufüllen.

Stimme überhaupt nicht zu 1	2	3	4	Stimme voll zu 5
◯	◯	◯	◯	◯

Begründung:

SUS

1. Ich denke, dass ich das System gerne häufig benutzen würde.

Stimme überhaupt nicht zu 1	2	3	4	Stimme voll zu 5
◯	◯	◯	◯	◯

2. Ich fand das System unnötig komplex.

Stimme überhaupt nicht zu 1	2	3	4	Stimme voll zu 5
◯	◯	◯	◯	◯

3. Ich fand das System einfach zu benutzen.

Stimme überhaupt nicht zu 1	2	3	4	Stimme voll zu 5
◯	◯	◯	◯	◯

4. Ich glaube, ich würde die Hilfe einer technisch versierten Person benötigen, um das System benutzen zu können.

Stimme überhaupt nicht zu 1	2	3	4	Stimme voll zu 5
◯	◯	◯	◯	◯

5. Ich fand, die verschiedenen Funktionen in diesem System waren gut integriert.

Stimme überhaupt nicht zu 1	2	3	4	Stimme voll zu 5
◯	◯	◯	◯	◯

6. Ich denke, das System enthielt zu viele Inkonsistenzen.

Stimme überhaupt nicht zu 1	2	3	4	Stimme voll zu 5
◯	◯	◯	◯	◯

7. Ich kann mir vorstellen, dass die meisten Menschen den Umgang mit diesem System sehr schnell lernen.

Stimme überhaupt nicht zu 1	2	3	4	Stimme voll zu 5
◯	◯	◯	◯	◯

8. Ich fand das System sehr umständlich zu nutzen.

Stimme überhaupt nicht zu 1	2	3	4	Stimme voll zu 5
◯	◯	◯	◯	◯

9. Ich fühlte mich bei der Benutzung des Systems sehr sicher.

Stimme überhaupt nicht zu 1	2	3	4	Stimme voll zu 5
◯	◯	◯	◯	◯

10. Ich musste eine Menge lernen, bevor ich anfangen konnte das System zu verwenden.

**Stimme überhaupt nicht zu 1**	**2**	**3**	**4**	**Stimme voll zu 5**
○	○	○	○	○

Dieser Fragebogen war verständlich.

**Stimme überhaupt nicht zu 1**	**2**	**3**	**4**	**Stimme voll zu 5**
○	○	○	○	○

Begründung:

Dieser Fragebogen war einfach auszufüllen.

**Stimme überhaupt nicht zu 1**	**2**	**3**	**4**	**Stimme voll zu 5**
○	○	○	○	○

Begründung:

Patient Interview for Pilot Study: Frozen Shoulder App

Description of User

Age:
Gender:
Start of frozen shoulder (month, year):
Further chronic diseases:

Smartphone-Usage
Do you own a smartphone?
Yes / No
If you do: Do you use your smartphone for

- o Phone calls and SMS
- o Social Platforms and messaging services (e.g., Facebook, WhatsApp, Skype)
- o Web browsing
- o Other apps
- o Fitness / Health-Apps

Quality Of Life / Coping

How have you been the last three weeks?

Have you used the app in the past three weeks?
Yes / No

➔ If you did not: Why?

Do you have the impression, that using the app has supported you in the past three weeks?

**Not at all**<br>**1**	**2**	**3**	**4**	**Very strong**<br>**5**
◯	◯	◯	◯	◯

TAM2

Intention to Use

3. Assuming I have further access to the app, I intend to use it.

**I do not agree at all**<br>**1**	**2**	**3**	**4**	**I totally agree**<br>**5**
◯	◯	◯	◯	◯

4. Given that I have access to the app, I predict that I would use it.

I do not agree at all<br>1	2	3	4	I totally agree<br>5
○	○	○	○	○

Perceived Usefulness

5. Using the app supports me (with respect to my shoulder disease).

I do not agree at all<br>1	2	3	4	I totally agree<br>5
○	○	○	○	○

6. Using the app increases the frequency of my shoulder training.

I do not agree at all<br>1	2	3	4	I totally agree<br>5
○	○	○	○	○

7. Using the app improves the accuracy of my shoulder training.

I do not agree at all<br>1	2	3	4	I totally agree<br>5
○	○	○	○	○

8. I find the app to be useful.

I do not agree at all<br>1	2	3	4	I totally agree<br>5
○	○	○	○	○

Perceived ease of use

5. The interaction with the app is clear and understandable.

I do not agree at all<br>1	2	3	4	I totally agree<br>5
○	○	○	○	○

6. Interacting with the app does not require a lot of my mental effort.

I do not agree at all<br>1	2	3	4	I totally agree<br>5
○	○	○	○	○

7. I find the app to be easy to use.

I do not agree at all<br>1	2	3	4	I totally agree<br>5
○	○	○	○	○

8. The app offers all relevant functionality.

I do not agree at all<br>1	2	3	4	I totally agree<br>5
○	○	○	○	○

Technical aspects:

What did you especially like?

What did you like not?

Did you change the view of the camera?
Yes/No/Why?
Have you read the exercise description text? Yes/No/Why?

If you did: did the audio version of the exercise text help you?
Yes/No/Why?
Have you use the mobility measurement with the smartphone sensors?
Yes/No/Why?
Would you like to document pain that occurred during measurement?
Yes/No
If yes: How?

What could we have done better during the study?

Do you think the personal instructions for app usage are necessary?
Yes/No

Exercises:

Have you the impression that you have correctly conducted the exercises?

Not at all<br>1	2	3	4	Yes<br>5
○	○	○	○	○

How could we improve the explanation of the exercises?

Could you perform the exercises? [The quality is assessed by a physiotherapist, misunderstandings are discussed]

Exercise 1:

No recollection<br>1	Mostly wrong, no effect of exercise<br>2	Partly wrong, some effect of exercise<br>3	Mostly correct, effect of exercise not impaired<br>4	Completly correct<br>5
○	○	○	○	○

Comment:

Exercise 2:

No recollection<br><br>1	Mostly wrong,<br>no effect of exercise<br>2	Partly wrong, some effect of exercise<br>3	Mostly correct, effect of exercise not impaired<br>4	Completly correct<br><br>5
◯	◯	◯	◯	◯

Comment:

Exercise 3:

No recollection<br><br>1	Mostly wrong,<br>no effect of exercise<br>2	Partly wrong, some effect of exercise<br>3	Mostly correct, effect of exercise not impaired<br>4	Completly correct<br><br>5
◯	◯	◯	◯	◯

Comment:

Exercise 4:

No recollection<br><br>1	Mostly wrong,<br>no effect of exercise<br>2	Partly wrong, some effect of exercise<br>3	Mostly correct, effect of exercise not impaired<br>4	Completly correct<br><br>5
◯	◯	◯	◯	◯

Comment:

USE-Fragebogen

Ease of Learning

I quickly learned to use the app.

I do not agree at all 1	2	3	4	I totally agree 5
◯	◯	◯	◯	◯

I easily remembered how to use the app.

I do not agree at all 1	2	3	4	I totally agree 5
◯	◯	◯	◯	◯

It is easy to learn to use the app (especially the mobility measurement with the smartphone sensors).

I do not agree at all 1	2	3	4	I totally agree 5
◯	◯	◯	◯	◯

I quickly became skillful using the app (especially the mobility measurement with the smartphone sensors).

I do not agree at all 1	2	3	4	I totally agree 5
◯	◯	◯	◯	◯

Satisfaction

I am satisfied with the app.

I do not agree at all 1	2	3	4	I totally agree 5
◯	◯	◯	◯	◯

I would recommend the app to other patients.

I do not agree at all 1	2	3	4	I totally agree 5
◯	◯	◯	◯	◯

The app is fun to use.

I do not agree at all 1	2	3	4	I totally agree 5
◯	◯	◯	◯	◯

The app works the way I want it to work.

I do not agree at all<br>1	2	3	4	I totally agree<br>5
◯	◯	◯	◯	◯

The app is wonderfull.

I do not agree at all<br>1	2	3	4	I totally agree<br>5
◯	◯	◯	◯	◯

I feel I need to have the app.

I do not agree at all<br>1	2	3	4	I totally agree<br>5
◯	◯	◯	◯	◯

The app is pleasant to use.

I do not agree at all<br>1	2	3	4	I totally agree<br>5
◯	◯	◯	◯	◯

The questions were easy to understand:

I do not agree at all<br>1	2	3	4	I totally agree<br>5
◯	◯	◯	◯	◯

Comments:

The questions were easy to answer:

I do not agree at all<br>1	2	3	4	I totally agree<br>5
◯	◯	◯	◯	◯

Comments:

SUS

11. I think that I would like to use the system frequently.

I do not agree at all<br>1	2	3	4	I totally agree<br>5
○	○	○	○	○

12. I found the system unnecessarily complex.

I do not agree at all<br>1	2	3	4	I totally agree<br>5
○	○	○	○	○

13. I thought the system was easy to use.

I do not agree at all<br>1	2	3	4	I totally agree<br>5
○	○	○	○	○

14. I think that I would need the support of a technical person to be able to use this system.

I do not agree at all<br>1	2	3	4	I totally agree<br>5
○	○	○	○	○

15. I found the various functions in this system were well integrated.

I do not agree at all<br>1	2	3	4	I totally agree<br>5
○	○	○	○	○

16. I thought there was too much inconsistency in this system.

I do not agree at all<br>1	2	3	4	I totally agree<br>5
○	○	○	○	○

17. I would imagine that most people would learn to use this system very quickly.

I do not agree at all<br>1	2	3	4	I totally agree<br>5
○	○	○	○	○

18. I found the system very cumbersome to use.

I do not agree at all<br>1	2	3	4	I totally agree<br>5
○	○	○	○	○

19. I felt very confident using the system.

I do not agree at all<br>1	2	3	4	I totally agree<br>5
○	○	○	○	○

20. I needed to learn a lot of things before I could get going with this system.

I do not agree at all<br>1	2	3	4	I totally agree<br>5
○	○	○	○	○

The questions were easy to understand:

I do not agree at all<br>1	2	3	4	I totally agree<br>5
○	○	○	○	○

Comments:

The questions were easy to answer:

I do not agree at all<br>1	2	3	4	I totally agree<br>5
○	○	○	○	○

Comments:
